# Knowledge, Attitude, and Perception of Cancer Patients towards COVID-19 in Pakistan: A Cross-Sectional Study

**DOI:** 10.3390/ijerph19137926

**Published:** 2022-06-28

**Authors:** Saadullah Khattak, Muhammad Faheem, Bilawal Nawaz, Maqbool Khan, Nazeer Hussain Khan, Nadeem Ullah, Taj Ali Khan, Rahat Ullah Khan, Kashif Syed Haleem, Zhi-Guang Ren, Dong-Dong Wu, Xin-Ying Ji

**Affiliations:** 1 Henan International Joint Laboratory for Nuclear Protein Regulation, School of Basic Medical Sciences, Henan University, Kaifeng 475004, China; saadullah271@gmail.com (S.K.); kakakhan3514@gmail.com (N.H.K.); 2 School of Life Sciences, Henan University, Kaifeng 475004, China; 3 District Headquarter Hospital Faisalabad, Punjab 38000, Pakistan; faheemagri@gmail.com; 4 Nishtar Institute of Dentistry Multan, Punjab 38000, Pakistan; drbilawal1@gmail.com; 5 Sino-Pak Center for Artificial Intelligence, Pak-Austria Fachhochschule: Institute of Applied Sciences and Technology, Haripur 22620, Pakistan; maqbool.khan@fecid.paf-iast.edu.pk; 6 Department of Microbiology, Tumor and Cell Biology, Karolinska Institute, 17177 Stockholm, Sweden; nadeemullah269@gmail.com; 7 Institute of Pathology and Diagnostic Medicine, Khyber Medical University, Peshawar 25100, Pakistan; tajalikhan.ibms@kmu.edu.pk; 8 Institute of Microbiology, Faculty of Veterinary and Animal Sciences, Gomal University, Dera Ismail Khan 29111, Pakistan; rahatullahkhan@gu.edu.pk; 9 Department of Microbiology, Hazara University, Mansehra 21120, Pakistan; kashifhaleem@hu.edu.pk; 10 Institutes of Traditional Chinese Medicine, Henan University, Kaifeng 475004, China; 11 School of Stomatology, Henan University, Kaifeng 475004, China

**Keywords:** COVID-19, cancer, knowledge, attitude, perception, Pakistan

## Abstract

Background: Cancer patients, being immunocompromised, are at higher risk of coronavirus disease (COVID-19). The current study determines cancer patients’ knowledge, attitude, perception, and impact of the COVID-19 pandemic. Method: A cross-sectional online survey was conducted in Pakistan from 1 April 2020 to 1 May 2020. The study respondents were cancer patients with ages equal to or greater than 18 years. Following a request for participation, the URL for the survey was distributed on numerous channels. Other social media platforms, including WeChat, WhatsApp, Facebook, Twitter, Instagram, Messenger, and LinkedIn, were used to increase cancer patient interaction. The questionnaire comprised five different sections such as: (1) sociodemographic information, (2) knowledge, (3) attitude, (4) perception, and (5) impact of COVID-19 on cancer patients. Descriptive medical statistics such as frequency, percentage, mean, and standard deviation were used to illustrate the demographic characteristics of the study participants. To compare mean knowledge scores with selected demographic variables, independent sample *t*-tests and one-way analysis of variance (ANOVA) were used, which are also practical methods in epidemiological, public health and medical research. The cut-off point for statistical significance was set at a *p*-value of 0.05. Results: More than 300 cancer patients were invited, of which 208 agreed to take part. The response rate was 69.33% (208/300). Gender, marital status, and employment status had a significant association with knowledge scores. Of the total recruited participants, 96% (n = 200) (*p* < 0.01) knew about COVID-19, and 90% were aware of general symptoms of COVID-19 disease, such as route of transmission and preventive measurements. In total, 94.5% (n = 197) (*p* < 0.01) were willing to accept isolation if they were infected with COVID-19, and 98% (n = 204) (*p* < 0.01) had reduced their use of public transportation. More than 90% (n = 188) (*p* < 0.01) of cancer patients were found to be practicing preventative measures such as using a face mask, keeping social distance, and avoiding handshaking and hugging. Around 94.4% (n = 196) (*p* < 0.01) of cancer patients had been impacted by, stopped or had changed cancer treatment during this pandemic, resulting in COVID-related anxiety and depression. Conclusion: The included cancer patients exhibited a good level of COVID-19 knowledge, awareness, positive attitude, and perception. Large-scale studies and efforts are needed to raise COVID-19 awareness among less educated and high-risk populations. The present survey indicates that mass-level effective health education initiatives are required for developing countries to improve and reduce the gap between KAP and COVID-19.

## 1. Introduction

Coronavirus disease (COVID-19) is a potentially fatal respiratory ailment caused by a newly discovered coronavirus named severe acute respiratory syndrome coronavirus 2 (SARS-CoV-2). The disease originated in the Chinese city of Wuhan and quickly spread throughout the mainland and beyond borders. On 11 March 2021, the World Health Organization (WHO) declared the COVID-19 pandemic [1]. As of 12 April 2022 (11:20 a.m., Islamabad, Pakistan time), 499,748,065 cases and 6,181,560 deaths have been reported worldwide due to COVID-19, with 1,525,666 cases and 30,361 deaths confirmed in Pakistan (https://coronavirus.jhu.edu/map.html, accessed on 12 April 2022). COVID-19 has a broad spectrum of clinical characteristics, ranging from asymptomatic, to mild symptoms, to severe illness, with an incubation period of 2–14 days. Common symptoms include coughing, fever, exhaustion, loss of smell or taste, shortness of breath, headache, sore throat, flu, vomiting, sickness, and diarrhea [2]. COVID-19 is mainly spread via respiratory droplets or direct contact; however, transmission via carriers has been examined [3]. COVID-19 is an extremely contagious virus. Isolation, proper hand-washing, social distance, a face mask, avoiding contact with the nose, eyes, and mouth, and avoiding crowded areas are just a few preventative measures designed to prevent the spread of this virus [2,4].

Numerous clinical risk factors are related to an increased risk of severe infection and death in COVID-19 patients, including a history of smoking, diabetes, cardiovascular disease, hypertension, respiratory disorders, age, and cancer [5,6]. Due to their immunocompromised state due to comorbidities and anticancer therapy, cancer patients are at a greater risk of COVID-19 infection. According to Onder et al., 20% of 355 patients with active cancer died due to COVID-19 [7]. Cohort research examining the clinical impact of COVID-19 on cancer patients discovered that cancer patients had a higher 30-day all-cause mortality [8]. According to data given by the WHO–China Joint Mission on Coronavirus Disease, the death rate for patients diagnosed with cancer and COVID-19 before 20 February 2020, was 7.6 per cent, which was significantly higher than the overall case fatality rate (3.8%) [9]. While this disease has caused considerable losses to communities worldwide, it has specifically proven to be devastating for vulnerable groups with pre-existing health issues, such as those diagnosed with breast cancer. Cancer’s immunosuppressive effects and the multimorbidity that commonly afflicts cancer patients [10] show that people who have breast cancer and other malignancies may be particularly vulnerable to COVID-19 infection [11]. Patients with an oncological history had a higher risk of requiring mechanical ventilation and admission to the intensive care unit when diagnosed with COVID-19, as well as a higher rate of death when compared with patients without cancer [12]. A recent nationwide, population-based study in Belgium found that solid cancer is an independent unfavorable prognostic factor for in-hospital death among COVID-19 patients [13]. In cancer, early detection and management are critical variables in determining survival results. Individuals with vague symptoms that may or may not represent malignant illness were understandably hesitant to seek treatment during a worldwide pandemic.

Cancer survivors may encounter adverse repercussions from the current epidemic, including delays in cancer detection and interruption of established treatment regimens (e.g., chemotherapy, radiation, surgery). After active treatment is over, all cancer survivors must attend annual checkups and have frequent scans, or yearly mammograms, which have been interrupted due to COVID-19. Treatment for estrogen-positive breast cancer can take up to 10 years from diagnosis, with endocrine therapies such as tamoxifen or aromatase inhibitors being used to reduce the risk of recurrence. As a result, women surviving breast cancer require ongoing access to healthcare services. A breast cancer diagnosis has been associated with debilitating physical and psychological complications during and after treatment [14,15].

Neurocognitive impairment is another common complication of breast cancer [16,17]. It may have a detrimental effect on a person’s mental health [18]. As a result, individuals diagnosed with cancer are more likely to experience clinical levels of emotional distress, manifesting as anxiety, sadness, or post-traumatic stress disorder [19,20,21]. As a result, determining the effect of the COVID-19 outbreak on cognitive and emotional health is critical for future assistance to the cancer community [19,20,21].

Global heterogeneity in the genomic structure of SARS-CoV-2 has a crucial role in the current outbreak of coronavirus illness COVID-19. According to research findings, the general root ancestry of global genomes varies depending on the extent of adaptability to the host [22]. Knowledge of citizens’ active roles in epidemics or pandemics is critical for appropriate planning and response, as their degree of knowledge, perceptions or misperceptions, behaviors, and trust may all impact the efficacy of activities and policies undertaken by health systems and authorities. Recognizing the importance of KAP surveys in COVID-19 management, several nations have begun to gather similar data at the national level [23,24]. Keeping the above concerns in mind, the current research was separated into two sections. First, we examined cancer patients’ knowledge, attitude, and perception (KAP) toward COVID-19. Second, we examined the effect of disruptions in scheduled oncology treatments (such as delayed treatment) and the Pakistani Government’s screening notice on emotional vulnerability and perceived cognitive function in a sample of cancer patients. Additionally, we examined the relationship between COVID-19-associated emotional vulnerability (COVID-EMV) and general anxiety, depression, and perceived cognitive function after controlling for rumination, worry, and other clinical and sociodemographic variables. Adherence to preventative measures is crucial for cancer patients, and their knowledge, attitude, and behavior regarding COVID-19 influence their compliance with these preventive measures. To help avoid infection, individuals must be educated about COVID-19 prevention procedures. Considering the preceding, we conducted a cross-sectional study to assess the KAP for COVID-19 among cancer patients in Pakistan and the impact of COVID-19 on cancer patients. The outcomes of this study will assist health authorities in creating effective strategies for educating and raising awareness among risk group populations.

## 2. Methods

### 2.1. Ethical Consideration

The Ethical Research Committee Department of Microbiology, Hazara University, Pakistan, approved the current study (study registration no: Micro/BC/2021/16).

### 2.2. Study Design and Procedure

From 1 April to 1 May 2020, an online/web-based research was planned through a link to Google form (https://docs.google.com/forms/, accessed on 2 May 2020) (Survey, Supplementary File S1). The first page of our web-based survey contained an informed consent page that explained the study to the participants. Participants were required to consent (or deny) participation to continue the survey. This was a voluntary survey for participation, and participants were provided with no incentive. The study’s subject population included cancer patients with only one age limitation, specifically, for greater than or equal to 18 years; there were no other exclusion criteria. A total of 300 people were invited, of which 208 agreed to take part. The response rate was 69.3% (208/300). According to Globocan2020, Pakistan’s reported population in 2020 was 220,892,332, with 178,388 new cancer cases, 329,547 5-year cancer cases, and 117,149 cancer deaths [25] (https://gco.iarc.fr/today/data/factsheets/populations/586-pakistan-fact-sheets accessed on 12 April 2022). Following a request for participation, the URL for the poll was distributed on numerous channels. Various social media including WeChat, WhatsApp, Facebook, Twitter, Instagram, Messenger, and LinkedIn, increased cancer patient interaction. The survey took approximately 10–15 min to complete. The repository data were only accessible to the core members, ensuring privacy.

## 3. Questionnaire Instruments and Variables

After evaluating the relevant scientific literature and published resources on COVID-19, a structured questionnaire was developed [11,23,26,27,28,29,30,31,32]. The questionnaire comprised five different sections, such as: (1) sociodemographic information, (2) knowledge, (3) attitude, (4) perception, and (5) impact of COVID-19 on cancer patients.

### 3.1. Sociodemographic Information

The characteristics section included the participant’s gender, age, level of education, marital status, cancer types, and employment status. Participants’ education responses were categorized into uneducated, school, college, graduate, and postgraduate. Similarly, the participant’s occupation was classified as retired, full-time, part-time, unemployed, worker, and housewife.

### 3.2. COVID-19 Related Knowledge

This section comprised the following items: COVID-19 knowledge, what COVID-19 is, how COVID-19 spreads, and COVID-19 symptoms. This included questions regarding different preventive strategies such as keeping a safe distance from someone who has symptoms; avoiding touching your eyes, nose, and mouth; refraining from shaking hands and hugging people; wearing face masks in public areas; and cleaning and disinfecting surfaces. Questions such as, “Do not assemble in groups”, “Avoid eating or drinking in public places”, and “Self-quarantine if unwell during the COVID-19 outbreak” were also included.

### 3.3. Perception Related to Universal Safety Precaution of COVID-19

The participants were asked seven questions to assess their understanding of COVID-19 and universal safety precautions. Each perception question was graded on a Likert-type scale with answers of “Yes,” “No,” and “Not sure.” The perception questionnaire collected responses from participants about their perceptions of several aspects of COVID-19, such as “COVID-19 symptoms appear in 2–14 days, during the outbreak”, “During the outbreak, eating well-cooked and safely handled meat can prevent the COVID-19”, “Sick patients should share their recent travel history with health care providers”, “Disinfect equipment and working area in wet markets at least once a day”, “Washing hands with soap and water can help in the prevention of COVID-19 transmission”, “I discussed COVID-19 prevention with my family and friends”, and “I reduced the use of public transportation”.

### 3.4. Attitudes

Three questions were asked about attitudes toward COVID-19 and agreement on the final control of COVID-19, such as the “people must take more care of each other now, and I will do everything to protect myself and my family”.

### 3.5. Impact of COVID-19 on Cancer Patients

A total of 17 questions were asked to measure the impact of COVID-19 in cancer patients. Each question on the impact scale was assessed using the Likert scale. The questions related to impact collected the participant’s information on the impact of different aspects of COVID-19, such as: Have you been obtaining cancer treatment this year (2021)? Since the COVID-19 epidemic began, have you had a reduction in work hours/pay? Have you had an increase in expenses? Have you not been able to pay for medications? Have you had trouble obtaining groceries? Have you stopped or changed your cancer treatment? Have you had an increase in child-care/elder-care responsibilities? Has any family member tested positive for COVID-19? How much has the COVID-19 epidemic impacted your ability to pay your monthly expenses? How much has the COVID-19 epidemic impacted your ability to pay for your cancer medications/treatment? Since the COVID-19 pandemic began, have you felt more socially isolated? Do you think the lockdown is increasing your psychological stress? Due to lack of physical activity, are you facing health issues?

### 3.6. Statistical Analysis

The collected participants’ responses through Google Forms were converted to Microsoft Excel and then exported into IBM SPSS v20 for Windows to perform prerequisite descriptive and statistical analyses. Descriptive medical statistics such as frequency, percentage, mean, and standard deviation were used to illustrate the demographic characteristics of the study participants. To compare mean knowledge scores with selected demographic variables, independent sample *t*-tests and one-way analysis of variance (ANOVA) were used, which are also practical methods in epidemiological, public health and medical research. The cut-off point for statistical significance was set at a *p*-value of 0.05.

## 4. Results

### 4.1. Study Participant’s General Characteristics

A total of 208 cancer patients from various regions of Pakistan participated in this study. Table 1 summarizes the sociodemographic characteristics of the study participants. A total of 51.52% of respondents were male, and 48.08% were female. Around 1.44% of participants were between 18 and 29, one-quarter (17.79%) were between 30 and 49, and 80.77% were above 50 (Supplementary Figure S1). The education levels were high school educated 8.2%, college educated 28.4%, graduate 49.5%, and postgraduate 10.6%. As illustrated in Supplementary Figure S2, only 3.4% of individuals were illiterate. Marital status data indicated that 93.75% of respondents were married, while 6.25% were unmarried. According to cancer types, most participants (38.94%) were diagnosed with breast cancer, followed by skin 24.52%, and lung cancer 19.23% (Supplementary Figure S3). Of the total, 31.25% of participants were retired, 28.85% worked full-time, 28.37% worked part-time, 3.84% worked, 2.40% were housewives, and 5.29% were unemployed (Supplementary Figure S4). The demographic characteristics of the study participants are summarized in Table 1.

### 4.2. COVID-19 Related Knowledge

Approximately 96.00% of participants were familiar with COVID-19. Each participant (100%) correctly identified COVID-19 as a viral disease. The majority of participants (98.5%) believed the disease was communicated through contaminated surfaces, through touching coins and banknotes (100%), through an asymptomatic person (92.8%), and airborne transmission (86.5%). In the section on measures to prevent disease spread, the majority of participants believed that proper hand-washing (83.2%), adequate social distance (99.5%), avoiding touching the eyes, nose, and mouth (99.5%), avoiding handshakes and hugs (98.5%), wearing face masks in public places (99.0%), avoiding sharing personal items (96.73%), and avoid eating and drinking in public places (97.5%), were all necessary. Additionally, 94.5% of respondents said that self-quarantine of sick patients was a preventative step against disease spread.

When asked about common COVID-19 symptoms, 98.5% of respondents identified fever, 99.0% dry cough, 99.5% body aches, 95.5% difficulty breathing, 87.0% sore throat, 94.7% diarrhoea, 90.8% tiredness, 96.0% loss of taste or smell, and 91.4% participants identified chest pain or pressure as common COVID-19 symptoms (Table 2 and Table 3).

### 4.3. Perception of Participants

The perception questionnaire elicited responses regarding participants’ perceptions of several features of COVID-19. In total, 99.5% of respondents stated that COVID-19 symptoms manifested within two to 14 days, 95.00% said that eating well-cooked meat and handling it safely could help prevent infection during the outbreak, 97.5% stated that sick patients’ recent travel history should be shared with health care providers, and 98.0% of respondents indicated that they have decreased their reliance on public transportation. There were significant differences in the correlation between perception and demographic characteristics associated with cancer patients (Table 3 and Table 4).

### 4.4. Attitude of Participants

Table 4 depicts the participants’ attitudes about COVID-19 prevention measures and their responses. Interestingly, most of the study participants had a positive attitude towards COVID-19. A total of 94.2% of participants thought that COVID-19 would be successfully controlled. About 91.8% of participants felt that people should take more care of each other during the pandemic. In addition, 98.0% of participants answered that they would do everything they could to care for themselves and their family members. Table 3 and Table 4 provides the details of these sections.

### 4.5. Impact of COVID-19 on Cancer Patients

Our survey showed that 2.88% of cancer patients did not obtain cancer treatment during this year, 92.75% had faced a reduction in work hours/pay, while 91.8% of cancer patients responded that they had an increase in expenses during the COVID-19 pandemic, 92.3% were not able to pay for their medication, and 85.6 % had trouble in obtaining groceries as shown in Table 5. Moreover, 94.2% of cancer patients responded that their treatment was stopped or changed during the COVID-19 pandemic, 88% answered that their child-care and elder-care responsibilities had increased, 89% of cancer patients’ had family members who had tested COVID-19 positive, 93.75% were impacted a little bit in paying for their monthly expenses, 73.56% were significantly impacted in paying for their monthly expenses. In contrast, 76.4% of cancer patients were not affected in being able to pay for their monthly expenses. However, 91.8% responded that they were concerned about paying for their medications/cancer treatment, while 0.5% thought they were not affected by their cancer medications/cancer treatment. About 92.7% believed they felt more socially isolated, 98.5% of cancer patients believed that the lockdown had increased their psychological stress, and 95.2% responded that they were facing health issues due to a lack of physical activity. Table 3 and Table 5 show the impact of COVID-19 on cancer patients.

## 5. Discussion

In our study, the cancer patients had good knowledge and awareness about COVID-19. In contrast to earlier research that revealed comparatively less awareness regarding COVID-19 disease transmission, the participants in this study had an excellent knowledge of COVID-19 [33,34,35,36]. Another study conducted in Pakistan showed that 99.6% of cancer patient were aware of COVID [37]. The participants’ better understanding of COVID-19 was due to frequent healthcare interaction, the observation of extra preventive measures taken by health professionals, and the government’s distribution of information via numerous sources.

Compared with the KAP of cancer patients in Nepal, a more significant number of cancer patients in Pakistan (vs 94.6% vs. 96.0%) were able to recognize the common symptoms of COVID-19 and had implemented preventative behaviors such as using a face mask (99% vs. 98.2%) [26]. By comparison, in a study conducted in Egypt, almost half of the patients (46%) had a good knowledge of COVID-19’s transmission, signs, and prognosis [38]. In Pakistan, a higher percentage of cancer patients (99.0%) use face masks than in Bangladesh (75.5%) [39]. Notably, almost all of the participants in our research used a face mask, and approximately 94.5% were willing to accept self-isolation if they were diagnosed with COVID-19. This shows a higher awareness in Pakistani people than in the Egyptian public (35% of participants were willing to wear a face mask, and almost 60% were willing to accept isolation if confirmed with COVID-19) [40]. This research paper’s high levels of positive practice and attitude might contribute to the study’s national scenarios, including the Health Ministry’s strict prevention strategies and the availability and accessibility of healthcare providers’ guidelines and recommendations.

Moreover, knowledge, education, interaction, or behavior, have changed effective communication channels throughout the country and the various healthcare sectors, including hospitals. It might also be because the participants were already familiar with COVID-19. However, the current study found that 2% of patients did not avoid drinking and eating in public places, and 1% were completely unaware of the importance of wearing masks.

In research by Yu et al., 1524 cancer patients in Chinese cancer centers were shown to have a two-fold increased risk of COVID-19 compared with the general population [41]. They believed that hospital visits played a role in the increasing prevalence. Furthermore, there was no clear rule on treating cancer patients based on their disease type, therapy type, or sub-population of cancer survivors (e.g., children, elderly) [42]. Due to the impending scarcity of healthcare resources and the heightened risk of anticancer therapy throughout this pandemic, making informed decisions on how and whether to offer cancer therapy was critical [43].

To cushion and shift resources to COVID-19 patients, the American College of Surgeons suggested delaying life-saving cancer operations and cancelling surgeries. This significantly impacted patients and may have resulted in the loss of critical chances in several resettable tumors [44]. According to the American Cancer Society Cancer Action Network, 24% of patients with cancer had their therapies delayed, and 12% were concerned about the uncertainty of future medicines. Urgent cancer referrals in the United Kingdom, which typically qualified for a two-week wait goal, were subjected to priority criteria created delays. Cancer screening programs were discontinued and were only available to symptomatic individuals [45]. In our study, 94.2% responded that due to the COVID-19 pandemic, their cancer treatment either stopped or changed. An amount of 97% of cancer patients responded that they had obtained cancer treatment in 2021, while 92.8% responded that since the COVID-19 pandemic, they had experienced reduced work and pay. An amount of 92.3% answered that they had not been able to pay for medications, and 85.6% had trouble obtaining groceries. In contrast, 88% of cancer patients responded that they had increased child-care/elder-care responsibilities during the COVID-19 epidemic. Furthermore, 92.7% of cancer patients felt more socially isolated, 98.5% thought that lockdown increased their psychological stress, and 95.2% thought they faced health issues due to lack of physical activity.

Related modelling research conducted in the United Kingdom considered a 2 week delay in cancer patient referrals and found an 84% decrease due to a 25% backlog of referrals caused by the lockdown. As a result, timely prioritizing of patients for whom referral delays will result in the loss of most life years should be evaluated [46]. Patients with particular cancers, such as leukemia, are also in danger from the pandemic. Fever is present in around 50.75% of patients with acute leukemia, putting them at risk of misdiagnosis. Other malignancies (mediastinal tumors or lung cancer) that present with respiratory symptoms, including cough, and test harmful for COVID-19, are also likely to be disregarded. In addition, stem cell transplant services are impacted by transplant risks, the number of procedures performed, and the presence of a matched donor. These circumstances might jeopardize a person’s chances of surviving [47].

According to research published by an international collaborative group studying the effects of COVID-19 on cancer therapy, cancer medical research projects such as clinical trials, often had to change their practices, as suspensions were on an increasing trend throughout the COVID-19 pandemic [48,49]. The World Health Organization [50] issued several specific guidelines, including implementing an isolate, test, treat, and track policy; hand washing after examining each patient; screening; and isolation of probable COVID-19 cancer patients in distinct wards. Furthermore, in cancer patients, care professionals were recommended to minimize the use of aerosol-generating procedures such as intubations [42]. The Centers for Disease Control and Prevention (CDC) advised high-risk persons to stay at home and avoid cruise ships and unnecessary air travel for cancer patients. They must, therefore, be prescribed treatment for several weeks.

Leaders in oncology care devised a strategy to deal with the Middle East respiratory syndrome coronavirus (MERS-CoV) problem in people with cancer during the MERS-CoV outbreak. Staff management, infection control, patient management, and a recovery plan are critical aspects of leadership, and communication is a significant component of this strategy. This strategy serves as a model for the COVID-19 pandemic and should be implemented to benefit immunocompromised people, such as cancer patients [51]. A recent Chinese experience in COVID-19 countermeasures in cancer suggests delaying elective surgery or adjuvant treatment for stable individuals and those with metastatic disease in endemic locations. If cancer patients, especially older people and those with additional comorbidities, are infected with SARS-CoV-2, strict and comprehensive monitoring should be suggested [31,52]. Masumi et al. advocated continuing therapy in patients with the curative aim in a special issue on their experience from the COVID-19 epicenter in the United States. Acute leukemias, for example, are a hematologic malignancy that require immediate attention and should be treated as such. Moreover, cellular immunotherapies and hematopoietic stem cell transplantation are life-saving treatments for most patients with severe illnesses and should not be postponed if feasible [43].

Consequently, the National Comprehensive Cancer Network (NCCN) [43] emphasizes the need to discuss supportive treatment with cancer patients if they contract COVID-19. With limited resources, oncologists must select which therapies are most likely to be effective and symptom-relieving, and which patients would benefit. Finally, oncologists must always be humane and avoid causing damage to patients’ premium non-Nocera. More than 98% of cancer patients in a similar study conducted during the pandemic period in Nepal were found to be responsible for compliance with suggested prevention strategies, and 94.6% were aware of the common symptoms of COVID-19 (fever, cough, sore throat, and shortness of breath), especially given the fact that formal education and literacy is at an all-time low [26]. Compared with Romanian cancer patients, Nepalese cancer patients had more knowledge and practice regarding COVID-19 [26,53].

Several studies on the relationships between KAP characteristics shed light on how public health policies might improve public health in emergencies, including new infectious disease pandemics, by implementing strategic intervention strategies. Furthermore, as our first example showed, information can play a critical role in improving the practice of public preventative behavior [26,54,55,56,57]. This finding suggests that information given via health actions to reduce and manage outbreaks should be based on evidence, and presented in simple language to increase public awareness of the issues [58]. Though it is hard to determine how often knowledge is required to achieve desired improvements in health outcomes, the effect of information on health behavior has been proven in a variety of public health settings [55,59,60]. They are predicated on the assumption that the general public may make “informed judgments” on health behavior by utilizing their knowledge of pertinent health problems. Whereas the term “informed decision-making” has many definitions [59,60], almost all believe that making informed decisions requires an adequate understanding of scientific facts about the relevant characteristics of the available options [61].

To provide adequate and exact information, efforts must be made to rectify inaccurate and misleading data. The term “infodemic” alludes to an oversupply of information, possibly false or damaging information disseminated via social media or other sources. The infodemic during the COVID-19 pandemic is a massive and ongoing challenge [62,63,64]. Since the pandemic, information creation and consumption have increased substantially, exposing the population to more misinformation [65,66]. When distributed health information clashes with existing cultural and system-based attitudes, engaging the public in behavior change campaigns may be severely hampered during health crises [67]. Rumors and falsehoods abound in all forms of communication [68]. We advise public medical professionals and authorities to improve knowledge and awareness alongside eliminating contextual issues that may obstruct the public’s ability to learn about health information. Importantly, this analysis found a high incidence of confusion about the source of infection by a few wild animals, with just 11.1% of respondents accurately responding that the information was inaccurate. The contexts of this misperception were not investigated in our study. As a result, we propose that future studies identify and track COVID-19-related misunderstandings across various communication channels to give correct, evidence-based information regarding the disease and prevention strategies. Furthermore, attitudes, particularly efficacy beliefs, have a significant and robust effect on performing protective factors, meaning that increasing COVID-19 prevention practices would involve public awareness and effectiveness perceptions. Evidence suggests self-efficacy is a crucial indicator of preventative behavior [69,70,71,72]. Our study revealed that even after receiving information, people must trust that preventive behaviors will be successful. To do and maintain behavior, people must believe that washing their hands will prevent them from becoming infected, rather than being informed. Although knowledge is at the core of learning, a mismatch between the information provided and received is expected [73]. According to public health specialists, health communication is a process driven primarily by individual cognitive and psychological characteristics. Our results suggest that boosting efficacy should be a priority. Therefore, COVID-19 behavior programmers might include message tactics that emphasize the efficacy of target behaviors (e.g., predicted decreased risks following hand hygiene practices) advocated by the programs. We further urge efforts to focus on people with poor efficacy views, especially those younger and less familiar with COVID-19.

Moreover, our findings revealed that sociodemographic characteristics influenced COVID-19 knowledge, attitudes, and practice. In particular, males and those with a lower education level have less understanding of COVID-19, making them especially vulnerable to the outbreak. Prior studies into the correlation between sociodemographic characteristics and knowledge level during the COVID-19 outbreak in China produced similar results [57,70], to Hong Kong [71], Pakistan [72] Malaysia [74] and Indonesia [75]. These studies in different countries revealed that the results differed among nations and were impacted by the types of human settlements. As a result, while creating health programs for COVID-19 and any future epidemics or pandemics, health and education agencies in various nations should strengthen their preventative measures by building specialized measures aimed at individuals in different settlements [76,77,78,79,80,81,82,83]. Knowledge disparity has been extensively studied in health communication. Many studies have sprung up in recent years, especially since the knowledge gap hypothesis proposes that people acquire knowledge at various rates, growing the knowledge gap with time, based on their socioeconomic situation, cognitive ability, and prior knowledge [84,85,86,87,88].

Although this study did not investigate the chronological trend of disparities, it did identify the gaps in all parameters within a correlation. Significant disparities in knowledge, attitudes, and actions were found among the participants. Therefore, minimizing knowledge inequities and prioritizing them with insufficient health information might help close health behavior and outcomes gaps. Those with deficient levels of COVID-19 knowledge should be given special attention, as they are less likely to have positive attitudes and engage in preventative activities. Policies and actions in the future should not be one-size-fits-all, since behavior elements are excessively dispersed throughout diverse social groups, as indicated in this study. We propose that health authorities’ analyses of fragile subgroups, priorities, strategies, and communication, struggle to fulfil the neglected demands, using a “person-centred” approach, not a “disease-centred” one.

This is one of several studies that have been undertaken to assess cancer patients’ knowledge, attitude, perception, and impact of the COVID-19 pandemic. It was an internet survey, so it was posted on social media; and many cancer patients in Pakistan were likely unable to see or access it. We inadvertently ignored the perspective of cancer patients who do not use the internet or social media. This investigation included several cancer patients. As the study was carried out during a lockdown, an online questionnaire was employed for evaluation. Another limitation of this research was that most of our responses were from cancer patients with internet access. Therefore, the remainder of cancer patients were omitted. However, the analysis may not have accurately reflected all of our current cancer patients’ proportions; nonetheless, it may demonstrate a general overview of the behavior present in cancer patients. Due to the small number of participants, further research such as this is needed to look at other aspects of COVID-19 in Pakistan. Our study was limited to participants who could communicate in English. Furthermore, the responses were based on honesty and were impacted by remembering capacity; thus, they may be biased recollections. We collected cancer patients using social media platforms instead of a representative sample from the entire country, so our findings may not be generalizable.

## 6. Reforms for the Welfare of Cancer Patients

The pandemic and accompanying prevention strategies also had a significant influence on the lives of cancer patients and their support networks [89]. Cancer patients and families typically face a significant deal of uncertainty about their future, which may be worsened by fears of contracting the virus, interruptions in their treatment, and the impact of social isolation [89]. Whereas the long-term implications of pandemic preventive actions and consequent treatment interruptions on tumor progression are unknown, the emotional burden on patients, families, and caregivers is becoming more apparent [90].

Furthermore, new versions of health services in the shape of telehealth and video consultations have evolved as a result of a confluence of variables such as physical distancing measures, patients’ unwillingness to visit healthcare centers, and initiatives to minimize demand on acute care services [91]. Throughout the epidemic, healthcare practitioners have been encouraged to provide virtual care, with the Australian Government proposing a series of new Medicare Benefits Schedule (MBS) item numbers—a list of government-subsidized services—for telehealth consultations throughout 2020 [92]. While telehealth had initially been used in a few settings, such as rural and remote communities [93] and specialist care [94], COVID-19 prompted a rapid scale-up (primarily of telephone-based telehealth) in less proven configurations, such as general practice, allied health, and hospital outpatient clinics [92].

## 7. Conclusions and Recommendations

Cancer patients, being immunocompromised, are at a higher risk of COVID-19. Increased mortality risk, missing early cancer detection possibilities, cancellation or cessation of life-saving medicines, distracting consequences, and diagnostic eclipsing are all impacts of this pandemic on cancer patients. The COVID-19 pandemic has impacted cancer patients’ care, including in-person visits, laboratory testing, imaging investigations, therapies, and operations. Moreover, the delay in cancer patients’ treatment has also increased their anxiety, depression, and worry. Masks, temperature checks, self-assessment questionnaires while visiting the hospital, and social distance are all new practices.

Consequently, assessing each therapy’s risk–benefit profile and other considerations is critical, including the patient’s financial situation and access to emergency services. There is also a pressing need to create successful rehabilitation strategies, emphasizing support services, notably psychological assistance. We may achieve active cancer survivors rather than passive victims by adopting innovative and effective COVID-19 management strategies and creating inventive approaches to provide cancer patients with ongoing therapy.

Generally, cancer patients who participated in our study had good knowledge and a positive attitude towards COVID-19. This suggests that health education programs designed to improve the knowledge of COVID-19 would encourage positive attitudes and safe practices. While the government takes significant steps to stop disease transmission, the other struggle must be to support the groups most affected by the disease’s economic consequences. In case of a vaccine or treatment approval for the disease, we urge it to be available at an affordable rate for developing countries. The government should control the use of the vaccine and treatment properly to preserve vulnerable and needy groups. Due to the low sample size, large-scale studies are recommended to scrutinize the country KAP towards COVID-19.

## Figures and Tables

**Table 1:** Demographic characteristics of participants (N = 208).

Characteristics		Number	Percentage (%)
Gender	Male	108	51.92
	Female	100	48.08
Age	18–29	3	1.44
	30–49	37	17.79
	Above 50	168	80.77
Marital Status	Single	13	6.25
	Married	195	93.75
Cancer Type	Breast	81	38.94
	Skin	51	24.52
	Lungs	40	19.23
	Liver	12	5.77
	Prostate	5	2.40
	Cervical	2	0.96
	Others	17	8.18
Employment Status	Retired	65	31.25
	Full-Time	60	28.85
	Part-Time	59	28.37
	Unemployed	11	5.29
	Worker	8	3.84
	Housewife	5	2.40

**Table 2:** Participant knowledge of COVID-19 (N = 208).

S. No.	Questions (Knowledge)	Yes n (%)	No n (%)	Not Sure n (%)
1	Possessed knowledge about COVID-19?	200 (96)	8 (4)	0
2	COVID-19 spreads by (Contaminated surfaces)	205 (98.5)	3 (1.5)	0
3	COVID-19 spreads by (Touching coins and banknotes)	208 (100)	0	0
4	COVID-19 spreads by (The disease could be transmitted from an asymptomatic person)	193 (92.8)	8 (3.8)	7 (3.4)
5	COVID-19 spreads by (Airborne transmission)	180 (86.5)	22 (10.6)	6 (2.9)
6	COVID-19 spreads by (Bite of animals)	170 (81.7)	23 (11.1)	15 (7.2)
7	Measures to prevent spread of the disease include (Proper hand washing)	173 (83.2)	20 (9.6)	15 (7.2)
8	Measures to prevent the spread of the disease include (Adequate social distance from a symptomatic individual)	207 (99.5)	1 (0.5)	0
9	Measures to prevent spread of the disease include (Avoiding touching eyes, nose and mouth)	207 (99.5)	1 (0.5)	0
10	Measures to prevent spread of the disease include (Avoiding handshakes and hugs)	205 (98.5)	2 ()	1 ()
11	Measures to prevent spread of the disease include (Putting on face masks in public places)	206 (99)	0	2 (1)
12	Measures to prevent spread of the disease include (Clean and disinfect surfaces)	202 (97)	3 (1.5)	3 (1.5)
13	Measures to prevent spread of the disease include (Avoid gathering in groups)	204 (98)	1 (0.5)	3 (1.5)
14	Measures to prevent the spread of the disease include (Avoid eating or drinking in public places)	203 (97.5)	2 (1)	3 (1.5)
15	Measures to prevent spread of the disease include (Self-quarantine if sick)	197 (94.5)	4 (2)	7 (3.5)
16	Common symptoms include (Fever),	203 (98.5)	2 (1)	3 (1.5)
17	Common symptoms include (Dry cough)	206 (99)	2 (1)	0
18	Common symptoms include (Body aches)	207 (99.5)	1 (0.5)	0
19	Common symptoms include (Difficulty in breathing)	198 (95.5)	2 (0.5)	8 (4)
20	Common symptoms include (Sore throat)	181 (87)	17 (8.2)	10 (4.8)
21	Common symptoms include (Diarrhea)	197 (94.7)	5 (2.5)	6 (2.8)
22	Common symptoms include (Tiredness)	189 (90.8)	11 (5.2)	8 (4)
23	Common symptoms include (Loss of taste or smell)	200 (96)	4 (2)	4 (2)
24	Common symptoms include (Chest pain or pressure)	190 (91.4)	10 (4.8)	8 (3.8)

**Table 3:** Association of knowledge, perception, attitude, and impact of COVID-19 with demographic variables of cancer patients (N = 208).

Variables	Categories	Knowledge Score (Mean ± SD)	t/f	*p*-Value	Perception Score (Mean ± SD)	t/f	*p*-Value	Attitude Score (Mean ± SD)	t/f	*p*-Value	Impact Score (Mean ± SD)	t/f	*p*-Value
Gender	Male	20.26 ± 0.86	3.20	0.01 (<0.05)	7.00 ± 0.00	2.818	0.01 (<0.05)	3.26 ± 0.00	4.166	0.01 (<0.05)	15.06 ± 1.237	0.599	0.01 (<0.05)
	Female	19.00 ± 3.85			6.65 ± 1.242			2.67 ± 0.792			14.74 ± 5.132		
Age	18–29	9.00 ± 11.356	40.487	0.01 (<0.05)	2.67 ± 3.786	68.120	0.01 (<0.05)	1.00 ± 1.732	65.491	0.01 (<0.05)	4.33 ± 7.506	66.941	0.01 (<0.05)
	30–49	18.22 ± 4.785			6.41 ± 1.363			2.27 ± 0.990			11.14 ± 5.822		
	Above 50	20.16 ± 0.704			7.00 ± 0.00			3.00 ± 0.00			15.92 ± 1.563		
Marital Status	Single	22.1538 ± 1.46	3.407	0.877 (>0.05)	7.00 ± 0.00	0.714	0.145 (>0.05)	3.26 ± 0.00	4.009	0.028 (<0.05)	13.00 ± 0.00	−7.565	0.007 (<0.05)
	Married	19.49 ± 2.79			6.82 ± 0.905			2.83 ± 0.589			15.03 ± 3.748		
Cancer Type	Breast	19.17 ± 3.331	1.25	0.283 (>0.05)	6.73 ± 0.962	2.253	0.040 (<0.05)	2.67 ± 0.758	3.184	0.05 (>0.05)	14.67 ± 5.104	2.065	0.059 (>0.05)
	Skin	20.29 ± 0.879			7.00 ± 0.00			3.00 ± 0.00			14.35 ± 1.092		
	Lungs	20.00 ± 0.0			7.00 ± 0.00			3.00 ± 0.00			15.85 ± 0.802		
	Liver	20.00 ± 0.0			7.00 ± 0.00			3.00 ± 0.00			17.00 ± 0.00		
	Prostate	20.00 ± 0.0			7.00 ± 0.00			3.00 ± 0.00			16.00 ± 0.00		
	Cervical	20.00 ± 0.0			7.00 ± 0.00			3.00 ± 0.00			17.00 ± 0.00		
	Others	18.82 ± 6.32785			6.24 ± 2.166			2.65 ± 0.996			13.41 ± 5.280		
Employment Status	Retired	20.00 ± 0.0	6.443	0.01 (<0.05)	7.00 ± 0.00	4.166	0.01 (<0.05)	3.00 ± 0.00	9.794	0.00 (<0.05)	15.31 ± 0.769	10.238	0.01 (<0.05)
	Full-Time	20.00 ± 0.0			6.41 ± 1.577			2.44 ± 0.970			12.93 ± 6.057		
	Part-Time	20.27± 4.903			7.00 ± 0.00			3.00 ± 0.00			16.75 ± 0.914		
	Unemployed	23.13 ± 0.647			7.00 ± 0.00			3.00 ± 0.00			13.00 ± 0.00		
	Worker	20.00 ± 0.641			7.00 ± 0.00			3.00 ± 0.00			13.00 ± 0.00		
	Housewife	19.65 ± 0.00			7.00 ± 0.00			3.00 ± 0.00			18.00 ± 0.00		

If the *p*-value was less than 0.05, then there was a difference between the knowledge of males and females.

**Table 4:** Perception and attitudes towards COVID-19 (N = 208).

S. No.	Questions (Perception)	Yes n (%)	No n (%)	Not Sure n (%)
1	Perceptions toward COVID-19 (COVID-19 symptoms appear in 2–14 days)	207 (99.5)	1 (0.5)	-
2	Perceptions toward COVID-19 ([During the outbreak, eating well-cooked and safely handled meat can prevent COVID-19)	198 (95)	6 (3)	4 (2)
3	Perceptions toward COVID-19 ([Sick patients should share their recent travel history with health care providers)	203 (97.5)	1 (0.5)	4 (2)
4	Perceptions toward COVID-19 (Disinfect equipment and working area in wet markets at least once a day)	203 (97.5)	1 (0.5)	4 (2)
5	Perceptions toward COVID-19 (washing hands with soap and water can help in the prevention of COVID-19 transmission)	200 (96)	1 (0.5)	7 (3.5)
6	Perceptions toward COVID-19 (I discussed COVID-19 prevention with my family and friends)	206 (99)	0	2 (1)
7	Perceptions toward COVID-19 (I reduced my use of public transportation)	204 (98)	2 (1)	2 (1)
	**Questions (Attitudes)**			
1	Attitudes (Do you agree that COVID-19 will finally be successfully controlled?)	196 (94.2)	9 (4.3)	3 (1.5)
2	Attitudes (It is important that people take more care of each other now)	191 (91.8)	5 (5.7)	12 (2.5)
3	Attitudes (I will do everything I can to protect my family and me)	204 (98)	3 (1.5)	1 (0.5)
	Average	197 (94.7)	5.6 (3.8)	5.3 (1.5)

**Table 5:** Participants’ views on COVID-19 impact.

S. No.	Questions (Impact)	Yes n (%)	No n (%)	Not Sure n (%)
1	Have you been obtaining cancer treatment this year (2020)?	202 (97)	3 (1.5)	3 (1.5)
2	Since the COVID-19 pandemic began, have you had a reduction in work hours/pay?	193 (92.8)	15 (7.2)	0
3	Since the COVID-19 pandemic began, have you had an increase in expenses?	191 (91.8)	17 (8.2)	0
4	Since the COVID-19 pandemic began, have you not been able to pay for medications?	192 (92.3)	16 (7.7)	0
5	Since the COVID-19 pandemic began, have you had trouble obtaining groceries?	178 (85.6)	30 (14.4)	0
6	Since the COVID-19 pandemic began, have you stopped or changed your cancer treatment?	196 (94.2)	12 (5.8)	0
7	Since the COVID-19 pandemic began, have you had an increase in childcare/eldercare responsibilities?	183 (88)	25 (12)	0
8	Any family member tested positive for COVID-19?	185 (89)	23 (11)	0
9	How much has the COVID-19 pandemic impacted your ability to pay your monthly expenses? (A little bit)	91 (43.8)	117 (56.2)	0
10	How much has the COVID-19 pandemic impacted your ability to pay your monthly expenses? (Not at all)	47 (22.6)	13 (6.2)	148 (71.2)
11	How much has the COVID-19 pandemic impacted your ability to pay your monthly expenses? (Very much)	10 (4.8)	11 (5.3)	187 (89.9)
12	How much has the COVID-19 pandemic impacted your ability to pay for your cancer medications/treatment? (Quite a bit)	153 (73.6)	15 (7.2)	40 (19.2)
13	How much has the COVID-19 pandemic impacted your ability to pay for your cancer medications/treatment? (Not at all)	159 (76.4)	16 (7.7)	33 (15.9)
14	How much has the COVID-19 pandemic impacted your ability to pay for your cancer medications/treatment? (A little bit)	13 (6)	13 (6)	182 (88)
15	Since the COVID-19 pandemic began, have you felt more socially isolated?	193 (92.7)	15 (7.3)	0
16	Do you think the lockdown is increasing your psychological stress?	205 (98.5)	3 (1.5)	0
17	Due to lack of physical activity, are you facing health issues?	198 (95.2)	10 (4.8)	0

## Data Availability

The datasets used and analyzed in the current study are available from the corresponding author on reasonable request.

## Supplementary Materials

The following supporting information can be downloaded at: https://www.mdpi.com/article/10.3390/ijerph19137926/s1, File S1: Survey (Knowledge, attitude, and perception of cancer patients towards COVID-19 in Pakistan: A cross-sectional study); Figure S1: Age-wise status of sample cohort; Figure S2: Education-wise response of cancer Patients; Figure S3: Cancer types wise response of cancer patients; Figure S4: Employment wise response of cancer patients.
