# Supplementary material for: Knowledge, Attitude, and Perception of Cancer Patients towards COVID-19 in Pakistan: A Cross-Sectional Study
Source: Int J Environ Res Public Health. 2022 Jun 28;19(13):7926. doi: 10.3390/ijerph19137926 (PMC9265320; doi:10.3390/ijerph19137926)
Supplement: Supplementary file 1 [file ijerph-19-07926-s001.zip › Figures Suppl.. file .pdf]

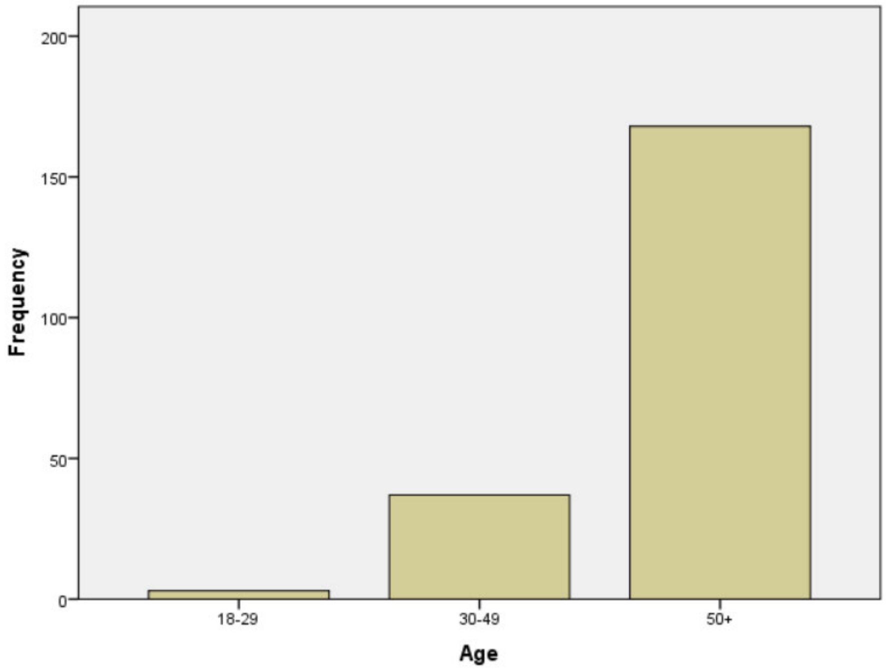

Figure S1: Age-wise status of sample cohort.

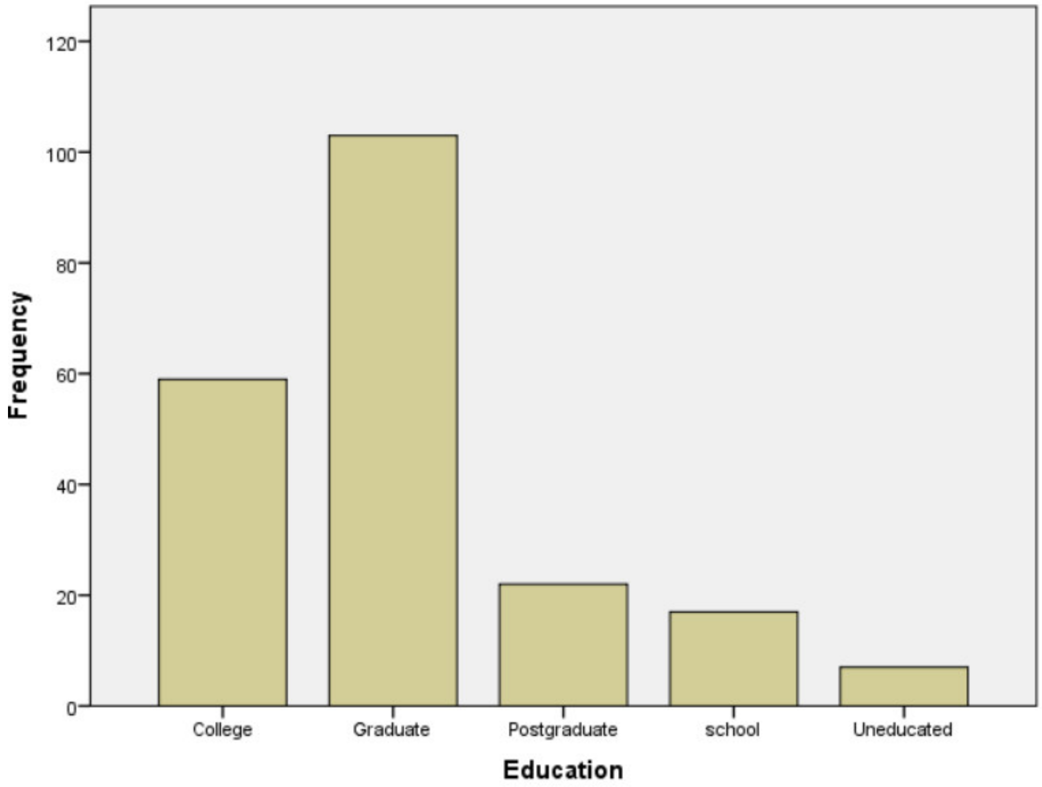

Figure S2: Education-wise response of cancer Patients

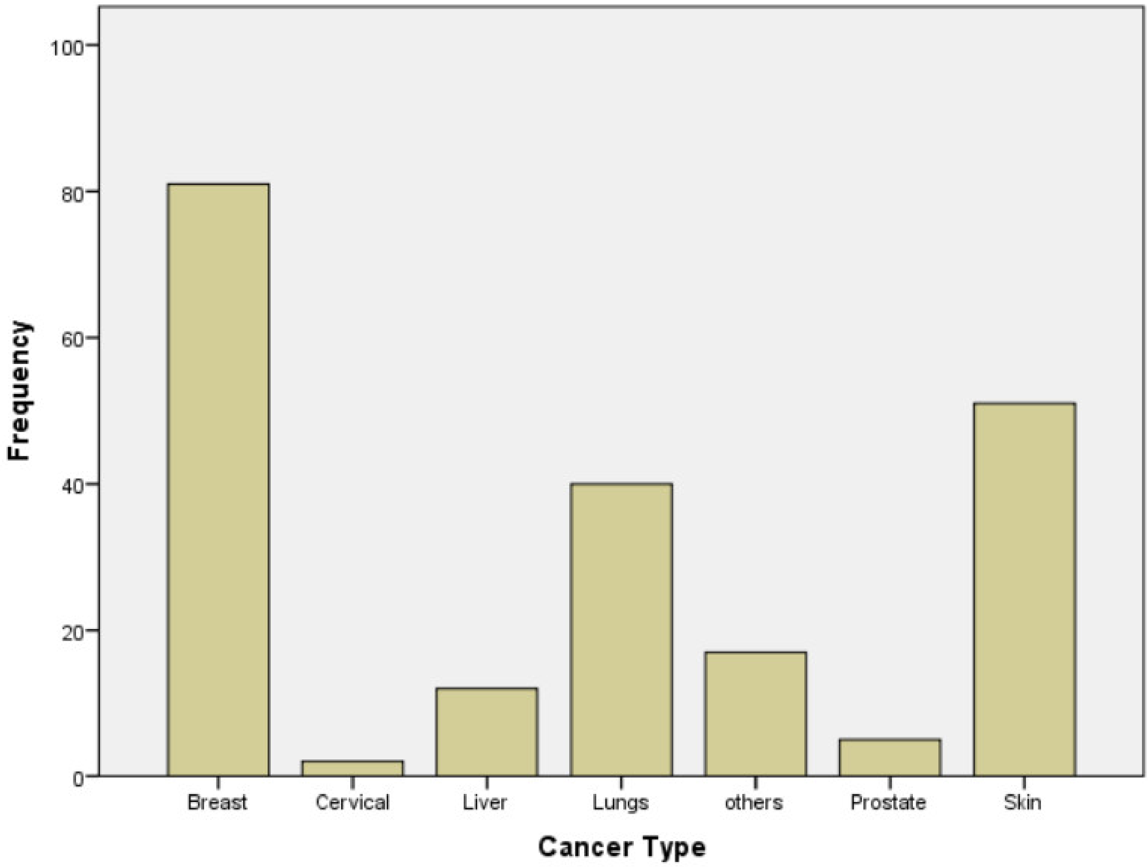

Figure S3:Cancer types wise response of cancer patients.

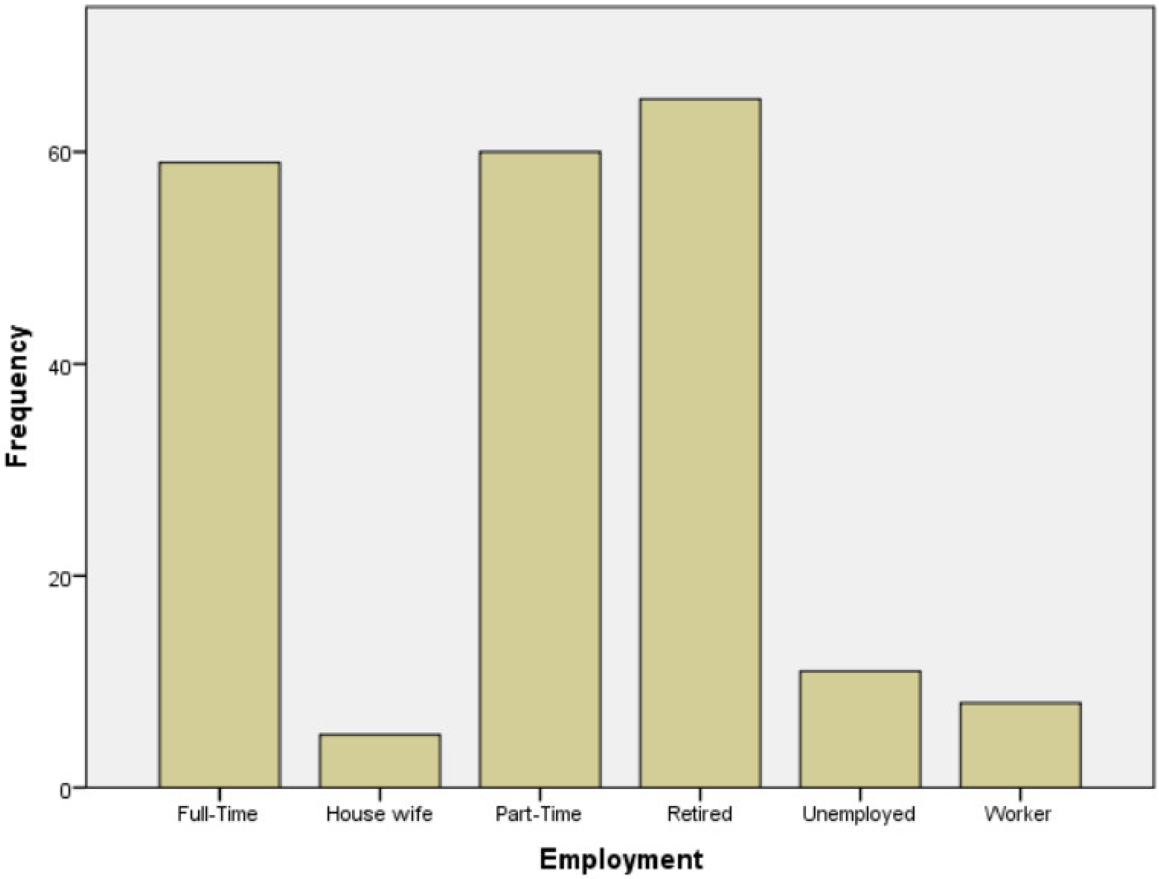

Figure S4:Employment wise response of cancer patients
