# Supplementary material for: Knowledge, Attitude, and Perception of Cancer Patients towards COVID-19 in Pakistan: A Cross-Sectional Study
Source: Int J Environ Res Public Health. 2022 Jun 28;19(13):7926. doi: 10.3390/ijerph19137926 (PMC9265320; doi:10.3390/ijerph19137926)
Supplement: Supplementary file 1 [file ijerph-19-07926-s001.zip › S1 Survey Knowledge, attitude, and perception of cancer patients towards COVID-19 in Pakistan.pdf]

Knowledge, attitude, and perception of cancer patients towards COVID-19 in Pakistan: A cross-sectional study

Informed consent	
**Hello Everyone, dear, we have conducted a survey titled "Knowledge, attitude, and perception of cancer patients towards COVID-19 in Pakistan: A cross-sectional study".** The results of the study will represent the Knowledge, attitudes and perceptions towards COVID-19 and Impact of COVID-19 on cancer patients. It is possible that the survey may take between 10-15 minutes to complete. You will not be asked for any personal information in the survey. Your personal information will be kept strictly confidential. You have the right to participate or deny, and you can withdraw from replying at any moment throughout your participation. The study will not benefit you financially or in any other way, but the findings may be considered by policymakers and lead to the implementation of COVID-19 treatments in Pakistan. Participants under the age of 18 should not take part in the survey.	
**Section 1: Socio-demographic information**	
1. Gender	○ Male<br>○ Female
2. Age	○ 18–29<br>○ 30–49<br>○ Above 50
3. Marital status	○ Single<br>○ Married
4. Cancer Type	○ Breast<br>○ Skin<br>○ Lungs<br>○ Liver<br>○ Prostate<br>○ Cervical<br>○ Others
5. Employment Status	○ Retired<br>○ Full-Time<br>○ Part-Time<br>○ Unemployed<br>○ Worker<br>○ Housewife
**Section 2: Knowledge**	
1. Possessed knowledge about COVID-19?	○ Yes<br>○ No<br>○ Not Sure
2. COVID-19 spreads by [contaminated surfaces]	○ Yes<br>○ No<br>○ Not Sure
3. COVID-19 spreads by [Touching coins and	○ Yes

banknotes]	○ No<br>○ Not Sure
4. COVID-19 spreads by [The disease could be transmitted from asymptomatic person]	○ Yes<br>○ No<br>○ Don't know
5. COVID-19 spreads by [airborne transmission]	○ Yes<br>○ No<br>○ Not Sure
6. COVID-19 spreads by [Bit of animals]	○ Yes<br>○ No<br>○ Not Sure
7. Measures to prevent spread of the disease include [Proper hand wash]	8. Yes<br>9. No<br>Don't know
8. Measures to prevent the spread of the disease include [adequate social distance from a symptomatic individual]	○ Yes<br>○ No<br>○ Not Sure
9. Measures to prevent spread of the disease include [Avoiding touching eyes, nose and mouth]	○ Yes<br>○ No<br>○ Not Sure
10. Measures to prevent spread of the disease include [Avoiding handshakes and hugs]	○ Yes<br>○ No<br>○ Not Sure
11. Measures to prevent spread of the disease include [Putting on facemasks in public places]	○ Yes<br>○ No<br>○ Not Sure
12. Measures to prevent spread of the disease include [Clean and disinfect surfaces]	○ Yes<br>○ No<br>○ Not Sure
13. Measures to prevent spread of the disease include [Avoid gathering in groups]	○ Yes<br>○ No<br>○ Not Sure
14. Measures to prevent the spread of the disease include [Avoid eating or drinking in public places]	○ Yes<br>○ No<br>○ Not Sure
15. Measures to prevent spread of the disease include [Self-quarantine if sick]	○ Yes<br>○ No

	○ Not Sure
16. Common symptoms include [Fever]	○ Yes<br>○ No<br>○ Not Sure
17. Common symptoms include [Dry cough]	○ Yes<br>○ No<br>○ Not Sure
18. Common symptoms include [Body aches]	○ Yes<br>○ No<br>○ Not Sure
19. Common symptoms include [Difficulty in breathing]	○ Yes<br>○ No<br>○ Not Sure
20. Common symptoms include [Sore throat]	○ Yes<br>○ No<br>○ Not Sure
21. Common symptoms include [Diarrhea]	○ Yes<br>○ No<br>○ Not Sure
22. Common symptoms include [Tiredness]	○ Yes<br>○ No<br>○ Not Sure
23. Common symptoms include [Loss of taste or smell]	○ Yes<br>○ No<br>○ Not Sure
24. Common symptoms include [Chest pain or pressure]	○ Yes<br>○ No<br>○ Not Sure
**Section 3:** Perception	
1. Perceptions toward COVID-19 [COVID-19 symptoms appear in 2-14 days]	○ Yes<br>○ No<br>○ Not Sure
2. Perceptions toward COVID-19 [During the outbreak, eating well-cooked and safely handled meat can prevent the COVID-19]	○ Yes<br>○ No<br>○ Not Sure

3. Perceptions toward COVID-19 [Sick patients should share their recent travel history with health care providers]	○ Yes<br>○ No<br>○ Not Sure
4. Perceptions toward COVID-19 [Disinfect equipment and working area in wet markets at least once a day]	○ Yes<br>○ No<br>○ Not Sure
5. Perceptions toward COVID-19 [Washing hands with soap and water can help in the prevention of COVID-19 transmission]	○ Yes<br>○ No<br>○ Not Sure
6. Perceptions toward COVID-19 [I discussed COVID-19 prevention with my family and friends.].	○ Yes<br>○ No<br>○ Not Sure
**Section 4:** Attitude	
1. Attitudes [Do you agree that COVID-19 will finally be successfully controlled?]	○ Yes<br>○ No<br>○ Not Sure
2. Attitudes [It is important that people take more care of each other now]	○ Yes<br>○ No<br>○ Not Sure
3. Attitudes [I will do everything I can to protect my family and me]	○ Yes<br>○ No<br>○ Not Sure
**Section 5:** Impact	
1. Have you been getting cancer treatment this year (2020)?	○ Yes<br>○ No<br>○ Not Sure
2. Since the COVID-19 pandemic began, have you had a reduction in work hours/pay?	○ Yes<br>○ No<br>○ Not Sure
3. Since the COVID-19 pandemic began, have you had an increase in expenses?	○ Yes<br>○ No<br>○ Not Sure
4. Since the COVID-19 pandemic began have you not been able to pay for medications?	○ Yes<br>○ No<br>○ Not Sure
5. Since the COVID-19 pandemic began, have you had trouble getting groceries?	○ Yes<br>○ No<br>○ Not Sure
6. Since the COVID-19 pandemic began, have you stopped or changed your cancer treatment?	○ Yes<br>○ No<br>○ Not Sure
7.Since the COVID-19 pandemic began, have you had an increase in childcare/eldercare	○ Yes<br>○ No

responsibilities?	○ Not Sure
8. Any family member tested positive for COVID-19?	○ Yes<br>○ No<br>○ Not Sure
9.How much has the COVID-19 pandemic impacted your ability to pay your monthly expenses? [A little bit]	○ Yes<br>○ No<br>○ Not Sure
10.How much has the COVID-19 pandemic impacted your ability to pay your monthly expenses? [Not at all]	○ Yes<br>○ No<br>○ Not Sure
11.How much has the COVID-19 pandemic impacted your ability to pay your monthly expenses? [Very much]	○ Yes<br>○ No<br>○ Not Sure
12.How much has the COVID-19 pandemic impacted your ability to pay for your cancer medications/treatment? [Quite a bit]	○ Yes<br>○ No<br>○ Not Sure
13.How much has the COVID-19 pandemic impacted your ability to pay for your cancer medications/treatment? [Not at all]	○ Yes<br>○ No<br>○ Not Sure
14.How much has the COVID-19 pandemic impacted your ability to pay for your cancer medications/treatment? [A little bit]	○ Yes<br>○ No<br>○ Not Sure
15. Since the COVID-19 pandemic began, have you felt more socially isolated?	○ Yes<br>○ No<br>○ Not Sure
16.Do you think the lockdown is increasing your psychological stress?	○ Yes<br>○ No<br>○ Not Sure
17. Due to lack of physical activity, are you facing health issues?	○ Yes<br>○ No<br>○ Not Sure
